# The Changes Over 15 Years Within a Pediatric Chronic Pain Service

**DOI:** 10.1002/pne2.70025

**Published:** 2026-05-06

**Authors:** Hadassah Buechner, Cameron Sanderson, Kit Min Chong, Amy Donaghy, Olivia Shand, Iona Lindsay, Mia Findlay, Ewan Wallace

**Affiliations:** ^1^ NHS Greater Glasgow and Clyde Glasgow Scotland UK; ^2^ University of Glasgow, College of Medical, Veterinary and Life Sciences Glasgow UK

**Keywords:** children, chronic pain, patient characteristics, patient outcomes, service evaluation

## Abstract

How do we know if a service is working? How has the presentation and management of children with chronic pain changed over the last fifteen years and what demonstrates adherence to changing guidelines? This audit of a large tertiary pediatric chronic pain service assessed relevant multivariate data gathered over the last 15 years to try to give an insight and answer these questions. All patients with electronic patient records (EPR) referred to the chronic pain service in the West of Scotland from 2010 to 2025 were audited. Any patients not seen in clinic at least once were excluded. Patients were grouped into epochs based on their most recent or last pain clinic appointment. 728 patients were included. The audit found that the service is seeing a greater proportion of patients with primary chronic pain diagnoses, accounting for over 40% of patients in the last epoch (*p* = 0.003). Significantly greater proportions of patients reported being bedbound (*p* < 0.001), needing walking aids (*p* = < 0.001) or having their activities of daily living being affected by pain (*p* = 0.05) in later epochs. The average number of referrals per patient, including referrals for physiotherapy and psychology increased. Although the average number of medications taken by patients increased, the proportion of patients taking gabapentinoids decreased (*p* = 0.180). This reflects adherence to current recommendations to reduce gabapentinoid prescribing, although the change was not statistically significant. There was a reduction in the percentage of patients discharged due to pain improvement and a greater proportion being transferred to adult services. The results suggest that patients are presenting with more severe complex pain, increasingly with no secondary cause. The service appears to be effective for approximately 30% of patients, who are discharged due to pain improvement. It is unknown how this compares to other services around the country.

## 1 Introduction

Chronic pain is defined as “an unpleasant sensory and emotional experience associated with, or resembling that associated with, actual or potential tissue damage” persisting continuously or intermittently for three months or longer [1]. The International Classification of Diseases (ICD‐11) further categorizes chronic pain into seven groups and can be viewed as broadly primary or secondary [2]. Within chronic primary pain, multiple subcategories exist [3]. This multidimensional classification reflects the heterogeneity of chronic pain and its diverse clinical presentations which can present as a challenge to manage [4, 5].

The impact of chronic pain in children and young people is profound, extending beyond physical symptoms to influence emotional wellbeing, social participation, family dynamics, and academic achievement [6, 7, 8, 9, 10]. At an individual level, chronic pain frequently reduces physical activity, impedes activities of daily living, and disrupts sleep [11, 12, 13, 14, 15]. Health‐related quality of life, encompassing physical, mental, and social wellbeing, is consistently lower in those with chronic pain compared to pain‐free individuals or those with acute pain, with mental health outcomes particularly affected [16, 17].

At the societal level, chronic pain generates a considerable healthcare and economic burden, although the cost arising from childhood pain is difficult to quantify [18]. In the UK, chronic back pain alone was estimated to cost between £5 billion and £10.7 billion annually [19]. Epidemiologically, chronic pain is both common and under‐recognized among children and adolescents. A recent systematic review of 119 studies, including over one million participants, reported a pooled prevalence of 20.8% [20]. The high prevalence, combined with the extensive consequences outlined above, underscores the urgency of optimizing recognition, management, and support pathways for young people with chronic pain.

Management of pediatric chronic pain is complex, reflecting its multifactorial nature and the interaction of biological, psychological, social, and family‐dynamic factors. Multidisciplinary care is considered best practice, often involving physicians, surgeons, nurses, psychologists, physiotherapists, occupational therapists, and other allied health professionals [21, 22]. Specialized pediatric pain services have been developed in some countries, but provision remains inconsistent and access is limited [23].

Pharmacological interventions play a smaller role in children than in adults, largely due to limited evidence of efficacy. Non‐opioid options (e.g., simple analgesics, topical agents, anticonvulsants, antidepressants, and non‐standard agents) may betrialed, but opioids are rarely indicated and should be reserved for older adolescents in cases refractory to other therapies. In contrast, non‐pharmacological approaches are increasinglyemphasized. These include not only physical therapies (e.g., exercise and manual therapy), psychological therapies (e.g., cognitive behavioral therapy and acceptance and commitment therapy), but also complementary or alternative interventions such as acupuncture or music therapy [24, 25].

Access to effective management in the UK remains inconsistent [26]. In addition, relatively little has been published to demonstrate achievable targets or benchmarks with reference to patient management strategies or outcomes in pediatric pain [18]. Evidence regarding the diagnostic pathways for chronic pain patients has shown high levels of variability, with limited implementation of current clinical guidelines [27]. Concerns regarding polypharmacy further complicate care [26, 28, 29].

This audit evaluates a Scottish tertiary center pediatric chronic pain service, using all available data over a 15‐year period. It seeks to describe the changes in the patient population, assess the extent to which improvements to the service have been implemented, while also examining patient management strategies and outcomes. In doing so, the audit will provide evidence to guide service improvement, identify weaknesses, and elicit discourse on what outcomes define an effective service.

## 2 Aims

1. Describe changes in presentations to the service, including the type of pain and demographics of patients in a Scottish tertiary pediatric pain center.
2. Analyze management strategies and compare this to growing guidance on management of chronic pain.
3. Review outcome data to identify areas for improvement and compare to existing literature.

## 3 Methods

### 3.1 Context

This audit, initiated in 2023 and continued until 2025, aimed to assess the pediatric chronic pain service at the Royal Hospital for Children in Glasgow. The pediatric chronic pain service is based in a tertiary center and receives referrals for all types of chronic pain, apart from headache, as patients are diverted to a separate headache clinic. Data was retrospectively collected from the electronic patient records (EPR) and every pediatric patient who attended the pain clinic was audited, from 2010 to 2023. Data was gathered from 2023 to 2025 prospectively as the intervention was taking place. The SQUIRE (Standards for Quality Improvement Reporting Excellence) guidelines were used to guide the reporting of this report.

### 3.2 Intervention

The project was conducted in sequential 5‐week blocks, with each block focusing on a specific dimension of either service provision or patient characteristics, including patient postcode as a proxy for financial deprivation, gender, neurodiversity, type of pain, surgical interventions, and psychological management. To ensure consistency and focus, groups of 3–5 medical students collected the data under supervision during these blocks.

### 3.3 Study of the Intervention

This quality improvement project used an audit–based approach to assess and improve service delivery. Inclusion criteria encompassed all patients who attended at least one pediatric pain clinic appointment. Relevant clinical variables were recorded and analyzed using Microsoft Excel.

A structured round‐table discussion was conducted with key service providers to explore potential improvements within the service. This resulted in a standardized clinic pro‐forma being introduced, alongside a renewed emphasis on the importance of documentation. This study also allowed for reflection on existing good practice and supported the identification of appropriate goals for service provision. The audit continued to 2025 to help evaluate the impact of the project on the variables collected.

### 3.4 Measures

Variables collected throughout the study included demographic data, management, including medications, surgeries, and referrals. Referrals were collected to include all referrals, including those that may be relevant to other health issues and not only pain. The aim was to provide a comprehensive view of the child's health experience. Patient outcomes were taken from reading the clinic letters. Adverse Childhood experiences (ACEs) were defined following Felitti et al. [30]. Financial deprivation was approximated using the Scottish Index of Multiple Deprivation (SIMD). SIMD is a measure that ranks areas from most to least deprived using indicators across domains such as income, employment, health, education, housing, crime, and access to services. Children's postcodes were gathered and they were grouped based on deprivation decile [31].

Neurodiversity describes individuals with variations in perception, cognitive processing, or communication [32] Neurodiversity was categorized in this audit as either autism spectrum disorder (ASD), attention deficit disorder (ADHD), awaiting assessment for ASD/ADHD, global developmental delay (GDD), learning difficulties, dyslexia, dyscalculia, dyspraxia, tic disorders, or Down syndrome. The term neurodiversity was chosen because it is transdiagnostic and describes clinically relevant phenotypes [33].

### 3.5 Analysis

Patients were grouped into epochs based on the date of their most recent pain appointment. Epochs were defined to ensure approximately comparable group sizes, to allow for ease of analysis.

- Epoch 1: 2010–2016, *n = 129*.
- Epoch 2: 2017–2019, *n = 139*.
- Epoch 3: 2020–2022, *n = 186*.
- Epoch 4: 2023–2025, *n = 274*.

Statistical analysis was performed in R version 4.5.1. Chi squared tests were used for categorical variables, and ANOVAs or t‐tests were used for continuous variables. Excel, R, and Microsoft Copilot were used for the generation of figures.

### 3.6 Exclusions

64 patients were excluded from this study. The exclusion criteria included: non‐attendance to the pain clinic appointment for any reason. If a patient attended at least one documented appointment, they were included. The reasons for exclusion are provided in Table S1.

### 3.7 Ethical Considerations

Patient data was anonymized for analysis. Only basic statistics were used to limit the transferability of the conclusions. This was an audit; no formal ethical approval was needed. Researchers followed ethical guidance as per the Caldicott principles and GPDR [34].

## 4 Results

### 4.1 Patient Characteristics

A total of 778 patients were included in the study. The patients were predominantly female (60%); this did not change across the epochs. Among patients with documented gender identity, 98% of patients identified as cisgender. Documentation of gender identity improved across epochs, with 21% of patients without gender identity documentation in 2010–2016 vs. 9.1% in the 2023–2025 cohort. The percentage of transgender (including non‐binary) patients increased across cohorts, from 2% in 2010–2016 to 4% in 2023–2025. The age of patients ranged from 10 months to 18 years. The median age decreased across epochs from 12 in 2010–2016 to 10 in 2023–2025 (*p* < 0.001). Across the whole sample, 26% of children were neurodivergent (*n* = 193), and the number increased each epoch from 19% (*n* = 25) in the first epoch to 34% (*n* = 93) in the last epoch.

### 4.2 Scottish Index of Multiple Deprivation (SIMD)

This audit involved patients across the entire range of the SIMD deciles, with a relatively even distribution of patients from all SIMD deciles in 2010–2016. The number of patients from the three most deprived deciles increased across the epochs, going from 35% of patients to account for 43% by 2023–2025. This change in descriptive values was not statistically significant (*p* = 0.34). The percentage of patients from the most affluent deciles [8, 9, 10] was 34% in the first epoch and 23% in the last epoch; there was no statistically significant change (*p* = 0.09). See Figure S1 for the breakdown.

#### 4.2.1 Pain Etiology

**FIGURE 1:**
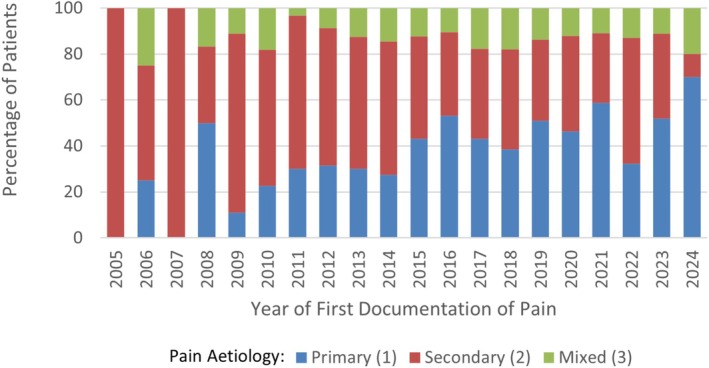
The type of pain presentation over time: N.B. an outlying value (*N* = 1) was excluded from year 2000 for clarity of interpretation.

Changes in pain etiology over time were analyzed using the date of first documentation of the pain which led to pain clinic referral, see Figure 1. Trends were analyzed using the Cochran‐Armitage test. This demonstrated an increase in a primary pain etiology over time (*p* < 0.001). The proportion of patients diagnosed with secondary pain decreased over time (*p* < 0.001), although diagnoses of mixed pain showed no clear trend over time (*p* = 0.57).

#### 4.2.2 ICD‐11 Pain Classifications

Chronic musculoskeletal pain was the dominant category across all epochs, increasing to over 50% by the final epoch (2023–2025). Neuropathic pain presentations peaked during the third epoch (2020–2022) at 18%, before declining thereafter. Post‐surgical or traumatic pain decreased from 2016 onwards but continued to account for a notable proportion of cases, at 18%. Cancer‐related pain remained relatively uncommon throughout, though it showed a slight increase in the final epoch (2023–2025). The breakdown of ICD‐11 classification is represented in graph form in Figure S1.

### 4.3 Management

#### 4.3.1 Pain Clinic Referral to Appointment Wait Times

Analysis of the waiting times between initial referral and a patient's first appointment demonstrated that the median waiting time remained stable between 70 to 90 days across all epochs. However, during one of the later epochs (2020–2022), a few extreme outliers were observed, with wait times extending to multiple years. These outliers inflated the mean waiting time despite the stable median. The proportion of patients seen within 3, 6, and 12 months remained consistent over time: ≥ 90% of patients were seen within 6 months, and ≥ 95% within 1 year. There was greater variability and longer waiting times in the last two epochs.

#### 4.3.2 Total Referrals

Across the entire cohort studied, 10 112 referrals were made. The average number of referrals per patient and number of GP referrals per patient were compared by ANOVA, with ad‐hoc two‐tailed *t*‐testing carried out between the means of the patients presenting between 2010 to 2016 and 2022 to 2025.

Across the periods 2010–2016, 2017–2019, 2020–2022, and 2023–2025, patients were referred an average of 9.74 times (min: 0, max: 63, SD: 9.21), 13.81 times (min: 0, max: 124, SD: 14.02), 14.60 times (min: 0, max: 70, SD: 11.60), and 15.42 times (min: 0, max: 82, SD: 12.59), respectively. Patients presenting during 2023–2025 had an average of 5.67 more referrals than patients presenting during 2010–2016 (*p* < 0.001).

#### 4.3.3 GP Referrals

Across the periods 2010–2016, 2017–2019, 2020–2022, and 2023–2025, patients were referred by their general practitioner to a secondary or tertiary service an average of 4.23 times *(min: 0, max: 26, SD: 4.92)*, 5.63 times *(min: 0, max: 32, SD: 6.05)*, 5.56 times *(min: 0, max: 22, SD: 5.15)*, and 5.88 times *(min: 0, max: 55, SD: 17.08)*, respectively. Patients presenting during 2023–2025 had an average of 1.64 more referrals from their GPs than patients presenting during 2010–2016 (*p* = 0.01).

#### 4.3.4 Referrals by Specialty

**FIGURE 2:**
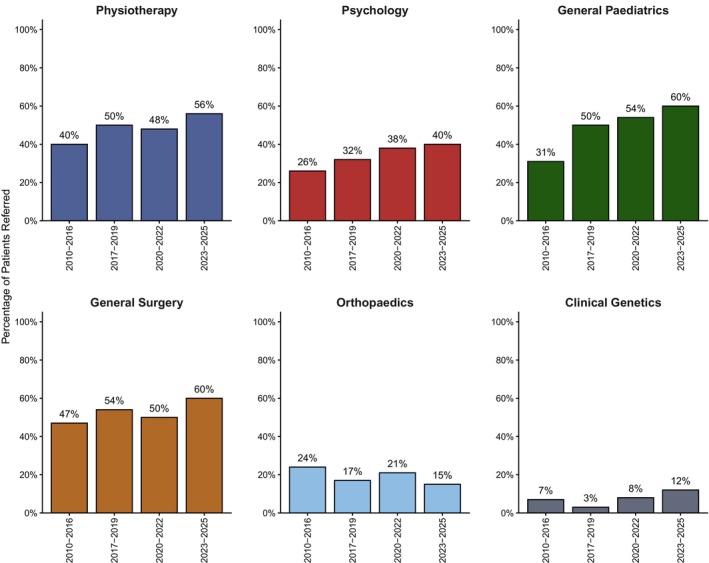
The proportion of patients in each epoch referred by any clinician to various specialties during their care.

Referrals sent on behalf of the patient by any clinician involved in their care wereanalyzed. Results for physiotherapy, psychology, generalpediatrics, general surgery, orthopedics, clinical genetics are shown. The trend of the percentage of patients referred to these services is illustrated in Figure 2 and Table 1. Across the periods 2010–2016, 2017–2019, 2020–2022, and 2023–2025 the percentage of patients referred to physiotherapy increased, 40%, 50%, 48%, and 56% respectively (*p* = 0.03); the percentage of patients referred to psychology increased 26%, 32%, 38%, and 40% respectively (*p* = 0.03); the percentage referred to general pediatrics increased, 26%, 32%, 38%, and 40% respectively (*p* = 0.03); the percentage of patients referred to general surgery decreased, 47%, 54%, 50% and 60% respectively (*p* = 0.04); the percentage referred to orthopedics did not significantly change, 24%, 17%, 21%, and 15% respectively (*p* = 0.11); the proportion of patients referred to clinical genetics increased, 7%, 3%, 8%, and 12% respectively (*p* = 0.01).

**TABLE 1:** The percentage of patients referred to a specialty to manage their pain by epoch.

Percentage referred to a Specialty	2010–2016 (%)	2017–2019 (%)	2020–2022 (%)	2023–2025 (%)	Significance
Physiotherapy	40	50	48	56	*p* = 0.03*
Psychology	26	32	38	40	*p* = 0.03*
General Pediatrics	26	32	38	40	*p* = 0.03*
General Surgery	47	54	50	60	*p* = 0.04*
Orthopedics	24	17	21	15	*p* = 0.11
Clinical Genetics	7	3	8	12	*p* = 0.01*

* significance was set at less than *p* = 0.05 and is noted in the far right column.

### 4.4 Referrals by Pain Type

Patients within time epochs were categorized by their pain type. Categories included primary, secondary, or mixed (evidence of primary and secondary pain syndromes) pain. Children with primary or secondary chronic pain, but not mixed pain, had significantly more total referrals in recent epochs compared to earlier epochs (*p* < 0.001). Only children with mixed chronic pain had significant increases in GP referrals over the epochs (*p* = 0.04). The details of changes in total referrals and referrals by GP are described in the Table S2.

#### 4.4.1 Primary Chronic Pain

Significant increases were observed for referrals to psychology and general pediatrics only. Across the time periods 2010–2016, 2017–2019, 2020–2022, and 2023–2025, the percentages of primary chronic pain patients referred to psychology were 22%, 32%, 40%, and 48% respectively (*p* = 0.03). Over the same time period, percentages of patients referred to general pediatrics were 38%, 49%, 58%, and 66% respectively (*p* = 0.01). All other specialties measured showed no significant change in the percentages of primary chronic pain patients referred to them. The figures relevant to the specialty referrals are shown in Figure S3a–c.

#### 4.4.2 Secondary Chronic Pain

Changes in the percentages of patients referred to psychology, generalpediatrics, general surgery, and clinical genetics were significant. Across the time periods 2010–2016, 2017–2019, 2020–2022, and 2023–2025, the percentages of patients referred to psychology were 27%, 33%, 36%, and 30% respectively (*p* = 0.01); the percentages of patients referred to general pediatrics were 28%, 55%, 50%, and 53% respectively (*p* < 0.00); the percentages of patients referred to general surgery were 51%, 60%, 50%, and 67% respectively (*p* < 0.001); and the percentages of patients referred to clinical genetics were 10%, 5%, 5%, and 18% respectively (*p* < 0.001). All other specialties measured showed no significant change in the percentages of patients referred to them.

#### 4.4.3 Mixed Chronic Pain

Only changes in the proportion of patients referred to clinical genetics were significant for this subgroup. For the time periods 2010–2016, 2017–2019, 2020–2022, and 2023–2025, the percentages of patients referred to clinical genetics were 0%, 0%, 7%, and 16% (*p* = 0.03), respectively. All other specialties measured showed no significant change in the percentages of patients referred to them.

### 4.5 Medications

An increased number of patients have been taking prescribed medications to manage their chronic pain between 2010 and 2025, see Figure S4a. The mean number of medications by year was 2.74 between 2010 to 2016; 3.17 in 2017–2019; 3.66 in 2020–2022; and 3.57 in 2023–2025. Children taking 8 or more medications were grouped for the purposes of calculating the means.

Most pain medications were prescribed more often, except for topiramate, which was prescribed for a consistently smaller proportion of patients. The type of medication broken down by year is shown in Figure S4b and Table S3. Gabapentinoids, amitriptyline, and topiramate prescriptions fell at a descriptive level over time, but only amitriptyline was significantly prescribed less (*p* = 0.013). Statistically significant increases in the use of NSAIDs (*p* = 0.027), paracetamol (*p* = 0.002), topical anesthetic (*p* < 0.001), and “other” medications (*p* = 0.003) were found. Use of gabapentinoids, opioids, melatonin, and topiramate did not show any statistically significant change.

**FIGURE 3:**
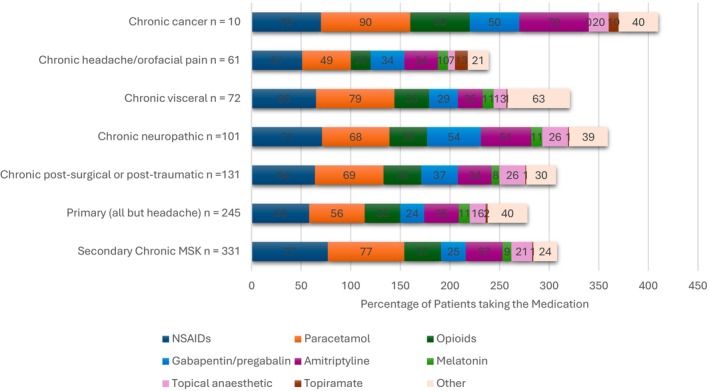
The type of medication taken based on type of pain. Note all primary pain types were collapsed into one category, apart from headache/orofacial pain; most patients with primary pain had primary MSK pain. The sample size for chronic cancer pain was only 10 patients, so this category should be interpreted with caution.

The type of medication taken was analyzed by pain type. The results demonstrated distinct medication signatures depending on the type of pain, see Figure 3 and Figure S5. A higher percentage of patients with neuropathic pain had prescriptions of gabapentin/pregabalin and, apart from cancer pain, also amitriptyline. Chronic headache/orofacial pain had the highest percentage of patients taking topiramate. Lower percentages of patients with primary pain syndromes were taking any medication.

### 4.6 Transcutaneous Electrical Nerve Stimulation (TENS)

**FIGURE 4:**
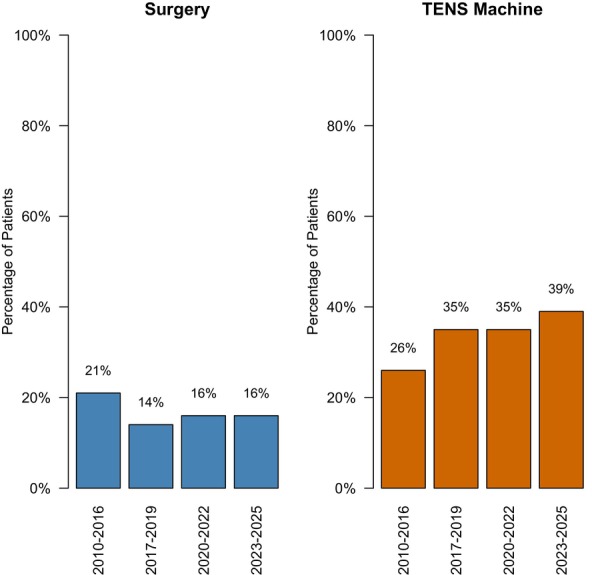
Percentage of patients requiring surgery or TENS for pain.

The number of TENS machines prescribed for pain increased slightly over time, see Figure 4. Between 2010 to 2016, 26% (*n* = 34) of patients were using a TENS machine, compared to 39% (*n* = 108) of patients between 2023 to 2025. The increasing proportions of patients using TENS machines were not statistically significant (*p* = 0.086).

### 4.7 Surgery

The percentage of pediatric patients requiring a surgical procedure as part of their chronic pain management reduced slightly on a descriptive level, see Figure 4. In 2010–2016, 21% (*n* = 27) of patients underwent a surgical procedure; compared to 14% (*n* = 19) between 2017 to 2019. The percentage increased slightly between 2017 to 2022, with 16% (*n* = 27) of the pediatric patients undergoing a surgical procedure to manage their pain between 2020 to 2022 and 16% (*n* = 44) requiring surgery between 2023 to 2025. The change in the proportion of patients requiring surgery over the years was not statistically significant (*p* = 0.423).

### 4.8 Functional Impact of Pain

#### 4.8.1 Emergency Department Attendances Related to Pain

Most patients had 0 to 1 attendances to the emergency department (ED) for pain. The median remained at 1 across the epochs. In 2020–2022, the mean rose to 2.8 attendances (compared with 2.2 in other epochs) and the number of patients with 5 or more attendances spiked to 22% of patients. In other epochs this accounted for 13%–15% of patients. The change in ED attendances over time was not significant (*p* = 0.28). There was a concentration of demand, where the top 10% of patients consistently accounted for more than half of all ED attendances. Figure S6 demonstrates the change in ED attendances.

#### 4.8.2 The Functional Impact of Pain

The percentage of cases noted as being bedbound currently or in the past due to their pain increased gradually from 12% to account for 13.5% of patients by 2025 (*p* < 0.001). The percentage of cases noted as requiring a walking aid currently or in the past remained stable at approximately 24%–28% in the years 2010–2022 with an increase to 33.2% in 2023–2025 (*p* < 0.001).

#### 4.8.3 Activities of Daily Living Affected by Pain

The percentage of cases reported as having Activities of Daily Living (ADLs) affected by pain increased over time, from 73.64% of cases in 2010–2016 to 86.13% of cases in 2023–2025. The percentage of cases noted as not having ADLs affected gradually decreased over time, from 7.75% in 2010–2016 to 4.38% in 2023–2025. ADLs affected by pain showed improved documentation over time. The rates of no documentation were noted to be 19% in 2010–2016 and fell to 9.5% in 2023–2025. The change in patients facing affected ADLs was not significant when excluding not‐documented cases (*p* = 0.20). When including “not documented,” the change was significant (*p* = 0.05). These trends reflect an improvement in documentation over the epochs, see Figure S7 and Table S4.

#### 4.8.4 Specific ADLs Affected by Pain

Whether pain had affected sleep, school attendance, exercise or socializing wasanalyzed. Over the epochs, 56% of children in 2010–2016 reported having their sleep affected. This proportion increased over time to account for 66% by 2023–2025. School attendance was impacted by pain. 54% of children of school attending age (5–16 years old) in the audit reported missing school due to pain. The median age of children who had school absences was 11, interquartile range 4.54% of school‐age children reported reduced school attendance in the first epoch; this increased to account for 57% of children in the most recent epoch. Exercise was the most reported ADL affected by pain, accounting for 65% of children in 2010–2016 and rising to 71% in 2023–2025. Socializing was least affected by pain, being reported by 31% of children in 2010–2016 and rising to 40% by 2023–2025. This increase in children reporting school and socializing being affected across the epochs was statistically significant (*p* = 0.0255 and *p* = 0.0243). The percentage‐wise increase in children reporting sleep and exercise being affected was not a statistically significant change (*p* = 0.0795 and *p* = 0.089).

### 4.9 Family Situation

**FIGURE 5:**
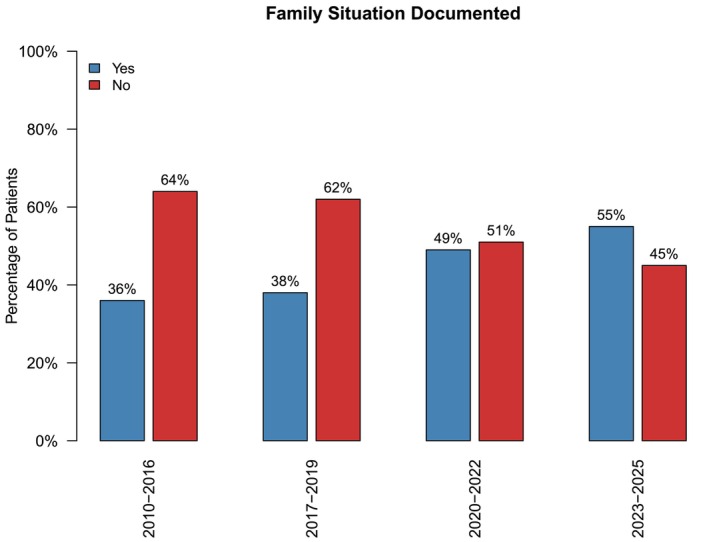
Documentation of family situation by epoch.

The percentage of patients who had their family situation documented steadily increased over the years, from 36% of cases documented for the 2010–2016 cohort to 55% of cases in the 2023–2025 cohort (*p* < 0.001), see Figure 5. There were no significant changes between the number of children with parents or siblings reported as having chronic pain between the epochs.

#### 4.9.1 Adverse Childhood Experiences and Bullying

The rates of no documentation were high ranging from 66% of children in 2020–2022 to 80% in 2017–2019. The data suggests that between 18%–15% of children had at least one or more ACEs. A few children in the sample had more than 5 ACEs, but the majority clustered between one or two documented ACEs. There were no clear trends over time in relation to the presence or type of ACEs. Rates of bullying gradually increased over the years, from 6% in 2010–2016 to 11% in 2023–2025. Rates of no documentation for bullying were also high, ranging from the highest of 79% in 2017–2019 to 73% in 2023–2025.

## 5 Patient Outcomes

### 5.1 Years Spent With Pain Service

Patients seemed to spend an increasing amount of time with the service. Possible outcomes noted included patient discharged, still in service, deceased or lost to follow‐up. The average time patients were in service rose from a mean of 1.96 years in the first epoch to 3.8 years in 2020–2022. The mean number of years in service for children last seen in 2023–2025 was 3. This change was statistically significant (*p* < 0.001), see Figure S8.

### 5.2 The Proportion of Patients Discharged

Across the time frame the proportion of patients being fully discharged from the service peaked in the first epoch 2010–2016 (*n* = 112, 87%) and saw a steady decline in the two consecutive epochs at (*n* = 116, 83%) and (*n* = 122, 66%) respectively. Only a third of the patients last seen in the 2023–2025 had been discharged (*n* = 81, 30%). A small percentage of patients continued their care with the service across three epochs. The main patient outcome in the last epoch was “still in service”, accounting for 57% of patients.

### 5.3 The Reason for Patient Discharge

**FIGURE 6:**
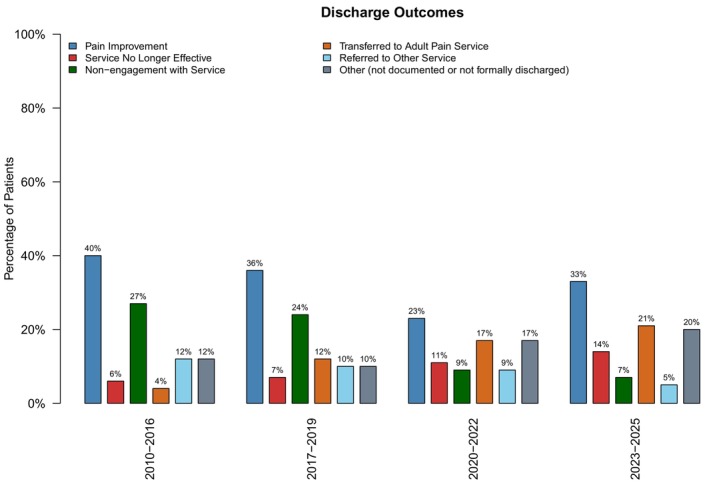
Reason for discharge.

Reasons for discharge included: pain improvement, the service no longer being effective, non‐engagement with the service by the patient, the patient being transferred to adults, or being referred to another service instead of the pain service. Discharges falling under other included not documented cases. The percentage of patients with different reasons for discharge is shown in Figure 6 and Table 2.

**TABLE 2:** Reasons for discharge.

Reason for discharge	2010–2016 (%)	2017–2019 (%)	2020–2022 (%)	2023–2025 (%)	Significance
Pain improvement	40	36	23	33	*p* = 0.74
Service not effective	6	7	11	14	*p* = 0.12
Non‐engagement	27	24	9	7	*p* = 0.006*
Transferred to adults	4	12	17	21	*p* = 0.003*
Referred to other services	12	10	9	5	*p* = 0.44
other	12	10	17	6	*p* = 0.18

The most frequent reason for discharge across all time periods was pain improvement, which was the main reason for discharge in the first epoch (40%). This percentage fell in subsequent epochs, decreasing to 36% in 2017–2019, then to 23% in 2020–2022. The fluctuation in percentage of patients discharged due to pain improvement was not statistically significant (*p* = 0.64).

Discharges due to non‐engagement decreased (2010–2016: 27%, 2017–2019: 24%, 2020–2022: 9%, 2023–2025: 7.4%). The decrease in discharges due to non‐engagement reached statistical significance (*p* = 0.004). Discharges due to service not being effective increased (2010–2016: 6%, 2017–2019: 7%, 2020–2022: 11%, 2023–2025:14%). This was not a statistically significant change (*p* = 0.12).

Children transferred to adult services followed a rising trend (2010–2016 4%, 2017–2019: 12%, 2020–2022: 17%, 2023–2025: 21%), which was significant (*p* = 0.003). Children were less likely to be referred to other pediatric services in the later years of the study (2010–2016: 12%, 2017–2019: 10%, 2020–2022: 9%, 2023–2025: 5%). This change was not statistically significant (*p* = 0.44).

## 6 Discussion

This audit analyzed data covering 728 patients in Scotland over 15 years. The aims were to assess any changes in presentation of pain, management strategies, and outcomes. The service was compared to growing guidance and other services' outcome reports on chronic pain in children [18, 35, 36]. The result of this work intends, through discussion and reflection, to drive further quality improvement strategies to improve chronic pain services inpediatrics.

### 6.1 Demographics and Presentation

The patient population was predominantly female (63%) and aged between 12 to 17 years of age. Most patients identified as cisgender, although the number of patients who identified as non‐binary or transgender increased steadily between 2010 to 2025. The higher proportion of female children with pain aligns with existing literature [37, 38, 39]. Girls are more likely than boys to experience musculoskeletal pain [40, 41], which was the most common pain presentation to the service.

The presence of non‐binary and transgender patients in the most recent epochs may be due to increasing awareness of the diverse spectrum of human gender which allows patients to find more accurate terms to describe their personal experience of gender [42]. Non‐binary and transgender youth are also known to be at an increased risk of developing chronic pain, which has been theorized to be due to earlier exposure to adversity due to the way they identify [43].

There was a transition away from secondary chronic pain predominance in clinic toward a more even share. Patients experiencing chronic primary pain rose from 27% to 41% by 2023–2025. Chronic musculoskeletal pain (primary or secondary) remained the dominant category throughout, increasing to over 50% by 2023–2025. Chronic primary pain has been defined as pain with no identifiable underlying cause, or pain that is disproportionate to any obvious injury or disease [35, 44]. The increase in people receiving a diagnosis of a chronic primary pain condition can be explained by increasing awareness in clinicians and the public. Over the past decade, chronic primary pain received a formal classification in ICD‐11, endorsement by the International Association for the Study of Pain, and incorporation into NICE guidelines [35]. Certain conditions, such as fibromyalgia, have had changes in diagnostic criteria, which has led to increased rates of diagnoses [45]. There is no published literature as to whether the increased rate of diagnosis is arising from primary or secondary care.

This paper showed that a relatively high proportion (30%–40%) of patients attending the pediatric chronic pain clinic were from the most deprived areas according to SIMD data. This likely reflects the broader population of the West of Scotland. According to the End Child Poverty Coalition, the average rate of child poverty across Scotland between 2023 to 2024 was 22% and in 2025, Glasgow City had the highest rates of child poverty at 36% [46]. Reports from England have noted inequalities with accessing tertiary care for chronic pain [47]. The audit data suggest that financial inequality is not limiting access to tertiary pain clinic care in the West of Scotland.

The percentage of neurodiverse patients appeared to increase between 2010 to 2025, accounting for over a quarter of patients in clinic across the whole sample audited. Neurodivergent children are known to have a higher incidence of chronic pain compared to their neurotypical counterparts, and as such neurodiversity is over‐represented in the pain management service [48]. The increasing percentage of neurodiverse people may suggest that the incidence of neurodiversity is increasing; conversely, it could be because of increased awareness of neurodiversity, where more children are being assessed and diagnosed [49].

### 6.2 Documentation of Social Factors

Although there was improvement in the documentation of social factors, the percentage of children whose EPR had no documentation was high. Consideration of the social factors which may be contributing to the pain presentation is important for identification of appropriate management strategies [50]. The audit aimed to advocate for increasing enquiry and documentation of social factors. The improvement in documentation noted could be due to the introduction of a clinic pro‐forma in 2023 (Table S5).

The rate of no documentation regarding ACEs was relatively high, and no trends were noted. This may be because ACEs are traumatic, and children may not want to discuss them with healthcare professionals with whom they are unfamiliar. It may also be that the extent of impact or significance of an adversity may only be evident later in the child's healthcare journey.

The most common ACEs noted in the audit were associated with the child's parents, and included divorce, bereavement or abuse. A prospective cohort study in Scotland found that witnessing parental mental health problems was experienced by 35% of children, witnessing parental separation was experienced by 32% of children, and corporal punishment was experienced by 22% of children [51]. The children often attend the pain management service with their parents and may not feel safe disclosing information about their traumatic experiences, which would affect documentation. This audit did not recommend routine screening for ACEs in the clinic pro‐forma [52, 53], but it was noted that many ACEs were disclosed when children were seen by clinical psychology.

### 6.3 How Services Are Responding to Pain

Children waited 1–3 months on average to be seen after referral. This wait time was much lower compared to 35 months as reported in Ireland [36]. This audit also found that children are spending longer with the pain clinic before being discharged. The average number of years in service rose and was mirrored by an increase in referrals per patient. This included both GP referrals, as well as referrals within secondary/tertiary care (Figure 2), with the average patient in 2023–2025 having more referrals than their counterpart in 2010–2016. Whilst an increase in referrals may reflect improved electronic documentation, the GP referrals which follow a different electronic pathway also demonstrated an increase over time.

Rates of medical comorbidity in children are rising [54, 55, 56]. Consequently, multi‐speciality input and multidisciplinary approaches may be more appropriate to manage children. The Royal College of Pediatrics and Child Health recommends the use of a multidisciplinary approach to provide integrated health care for children and young people [23, 57]. The increase in referrals may reflect more comprehensive healthcare for patients. For example, rates of referrals to physiotherapy and psychology increased significantly (Figure 2). Although increasing multi‐disciplinary input can be seen as an improvement, it is likely that children and families are becoming overwhelmed with appointments [58, 59, 60, 61]. Pain services may need to consider restructuring to reduce appointment burden [62, 63, 64].

### 6.4 Pain Management

The mean number of medications taken by the children increased. An increase in medicines prescribed for pain suggests an increasing reliance on pharmacological methods to manage chronic pain. This may lead to overmedicalization of pain in children [50, 65, 66, 67]. Furthermore, there is less evidence to suggest that pharmacological management is as effective at managing chronic pain in pediatric patients compared to the adult population [68, 69, 70].

There was a decrease, at a descriptive level only, of the prescription of anticonvulsants such as gabapentin and pregabalin. The prescription of these medications may reflect the service's attempts to deprescribe and reduce the prescription of gabapentinoids in line with current guidance [71]. The advice arises from safety concerns regarding gabapentinoids and increasing mortality rates in those on long‐term gabapentinoids.

A reduction in prescribing opioids for chronic pain in children and young people is imperative [28, 72]. The audit did not find a significant reduction in opioid prescribing, although a lower percentage of patients taking opioids was found in the most recent epoch. Patients undergoing surgery for pain often had opioids prescribed post‐operatively, and this audit found a non‐significant reduction in the average number of operations for chronic pain over time. Evidence from the United States suggests that many children do not use their prescribed post‐operative opioid medication, and that non‐opioid post‐operative prescriptions may be safe and acceptable [73, 74, 75, 76]. Safer alternatives to opioids should be emphasized in surgical settings.

A non‐significant but descriptive increase in the use of transcutaneous electrical nerve stimulation (TENS) machines between 2010 and 2025 was found. There is moderate evidence to suggest that this therapy is effective in providing analgesia for chronic musculoskeletal pain in adults [77, 78]. The increase in the use of TENS could reflect a growing appetite to trial alternative therapies for chronic pain, despite a lack of data for the efficacy of TENS in children.

### 6.5 The Severity and Impact of Pain

The severity of pain, measured by the percentage of patients reporting using walking aids, being bedbound or having other ADLs affected, showed statistically significant increases over the epochs. This could suggest that children are presenting with worse pain than before, consistent with epidemiological literature [79, 80, 81, 82]. Or it could be hypothesized that there has been a change in how patients, families and society deal with chronic pain. Pain behavior refers to how individuals respond to pain, communicate pain and seek help for pain. Avoidance of activities of daily living, usage of walking aids and remaining in bed are all behavioral responses to pain. It is possible that the changes noted in these variables are associated with changes in pain behaviors in recent years [83, 84].

### 6.6 Patient Outcomes

There was a decreasing proportion of patients discharged from the service over the epochs. In the most recent epoch (2023–2025), the apparent decline in the length of years that patients are in the service for (Figure S8) is best explained by incomplete outcome data. Most patients (57%) are still with the service, which introduces right‐censoring or cut‐off bias. Discharge rates were inflated during the 2010–2016 period. This could be due to the predominance of secondary chronic pain types, where patients were assessed, treated, and discharged relatively quicker than patients with primary pain. So far, little evidence suggests a difference in prognosis between primary and secondary pain types [85, 86].

The audit noted a significant rise in percentages of patients transferring to adult services, possibly due to increasing emphasis on continuity of care through the implementation of the Scottish National Pain Management Programme in 2015 [87]. Pain services were thought to be a temporary intervention with discharges following medication prescription but have now transitioned to longer‐term holistic care models [72, 88].

### 6.7 Discharges Due to Pain Improvement and Non‐Engagement

The most common reason for discharge across all time periods was pain improvement. Discharge due to pain improvement declined markedly after 2017, reaching its lowest point in 2020–2022 (23%), see Figure 6. This reduction may be linked to factors such as lost follow‐ups and rebooking difficulties during the COVID‐19 pandemic, when service disruption and reduced in‐person consultations limited care delivery [89–91]. This assumption is supported by the partial rebound in 2023–2025, when discharges due to pain improvement rose to 33%. The percentage of discharges due to non‐engagement saw a sharp reduction. The improvement is likely due to the implementation of text‐based appointment reminder systems and the option for telephone/video consultations throughout the NHS.

### 6.8 Strengths and Limitations

This audit is one of the first to analyze trends in pediatric chronic pain across 15 years. Comprehensive data covering presentation, management, outcomes, and social factors were gathered. The data contributes to service evaluation and outcome reporting and can be used to suggest benchmarks for what constitutes an effective pain service.

A key limitation to this audit is the unequal distribution of the number of years within each epoch, specifically the first epoch (2010–2016) which accounts for a substantially longer duration than subsequent epochs. Epochs were defined to achieve sample sizes to facilitate statistical analysis, although this introduced temporal heterogeneity between the groups.

Over a span of a longer duration, the aspects of service provision such as referral pathways, patient documentation, and healthcare policies may have evolved considerably, which increases the risk of information bias. As a result, temporal comparisons between the first epoch and later epochs may miss finer changes in the first years and may make the analysis less valid. Future audits should incorporate fixed‐duration epochs to allow for more direct comparisons over time.

The nature of electronic patient records (EPR) was the biggest limitation to this audit. For example, due to a lack of documentation on EPR, the rate of obesity in the pediatric sample was not gathered. Obesity is a risk factor for musculoskeletal pain (92–94). The rate of childhood obesity has been increasing (95). Due to a lack of data, the percentage of overweight or obese children in the clinic could not be identified. Furthermore, not all referrals were documented in EPR. All patients included in the audit were taken from the pain clinic's referral database. Despite this, documented referrals on EPR for the pain service did not encompass 100% of the patients included. The referral results should be interpreted with caution, since the proportion of patients referred to the pain management services based on EPR increased over the epochs 71%, 83%, 88%, and 94% respectively (*p* < 0.001). *This* reflects better electronic documentation, as each patient should have had a documented referral.

## 7 Conclusion

This detailed audit reported on the trends in children presenting to a tertiary chronic pain service. The results demonstrated an increase in the number of children presenting with primary chronic pain. The number of referrals per child to multiple different specialties also increased. In addition, the pain reported by the children appears to be more severely impactful than in the past, with more children reporting using walking aids or being bedbound. Improved documentation of certain social factors highlights the success this audit had for raising the importance of psychosocial factors to the local team.

The changes in management reflect adherence to recent guidance to reduce the prescription of gabapentinoids in children. Despite this, the average number of medications taken per child has increased. Concerningly, the proportion of patients discharged because of pain improvement has fallen. The results suggest that children may be presenting with more complex, severe, or treatment refractory pain. Due to the absence of globally agreed benchmarks for effective pain management and pain remission in children, it is not possible to conclude whether the service is performing well in comparison to other services. Future work should aim to create a consensus on what defines an effective pediatric chronic pain service.

## Conflicts of Interest

The authors declare no conflicts of interest.

## Supporting information


**Figure S1:** SIMD distribution.
**Figure S2:** ICD‐11 classification for all patients. Patients with only headache and orofacial pain were seen between 2010 to 2013, and after that all these patients were diverted to a headache clinic.
**Figure S3a:** Significant changes in referrals noted in Primary Chronic Pain Patients.
**Figure S3b:** Significant changes in referrals noted in Secondary Chronic Pain Patients.
**Figure S3c:** Significant changes in referrals noted in Mixed Chronic Pain Patients.
**Figure S4a:** Mean number of medications taken by pediatric patients between 2010 to 2025.
**Figure S4b:** The percentage of patients within an epoch taking a certain medication.
**Figure S5:** Heatmap of medication usage by pain type.
**Figure S6:** Percentage of patients with ED attendances greater than or equal to 5.
**Figure S7:** Functional Impact of Pain by Year.
**Figure S8:** Number of years patients spent with the pain service.
**Table S1:** Reasons for exclusion.
**Table S2:** Referral data by pain type.
**Table S3:** Proportion of patients taking medication for pain. Examples of other medications included: diazepam, lamotrigine, hyoscine butylbromide, baclofen.
**Table S4:** Documentation of ADLs affected by Pain.
**Table S5:** pain clinic pro‐forma.

## Data Availability

The data that support the findings of this study are available on request from the corresponding author. The data are not publicly available due to privacy or ethical restrictions.
