# Supplementary material for: The Changes Over 15 Years Within a Pediatric Chronic Pain Service
Source: Paediatr Neonatal Pain. 2026 May 6;8(2):e70025. doi: 10.1002/pne2.70025 (PMC13147218; doi:10.1002/pne2.70025)
Supplement: Supplementary file 1 — Figure S1: SIMD distribution. Figure S2: ICD‐11 classification for all patients. Patients with only headache and orofacial pain were seen between 2010 to 2013, and after that all these patients were diverted to a headache clinic. Figure S3a: Significant changes in referrals noted in Primary Chronic Pain Patients. Figure S3b: Significant changes in referrals noted in Secondary Chronic Pain Patients. Figure S3c: Significant changes in referrals noted in Mixed Chronic Pain Patients. Figure S4a: Mean number of medications taken by pediatric patients between 2010 to 2025. Figure S4b: The percentage of patients within an epoch taking a certain medication. Figure S5: Heatmap of medication usage by pain type. Figure S6: Percentage of patients with ED attendances greater than or equal to 5. Figure S7: Functional Impact of Pain by Year. Figure S8: Number of years patients spent with the pain service. Table S1: Reasons for exclusion. Table S2: Referral data by pain type. Table S3: Proportion of patients taking medication for pain. Examples of other medications included: diazepam, lamotrigine, hyoscine butylbromide, baclofen. Table S4: Documentation of ADLs affected by Pain. Table S5: pain clinic pro‐forma. [file PNE2-8-e70025-s001.docx]

*Supplementary Figures*


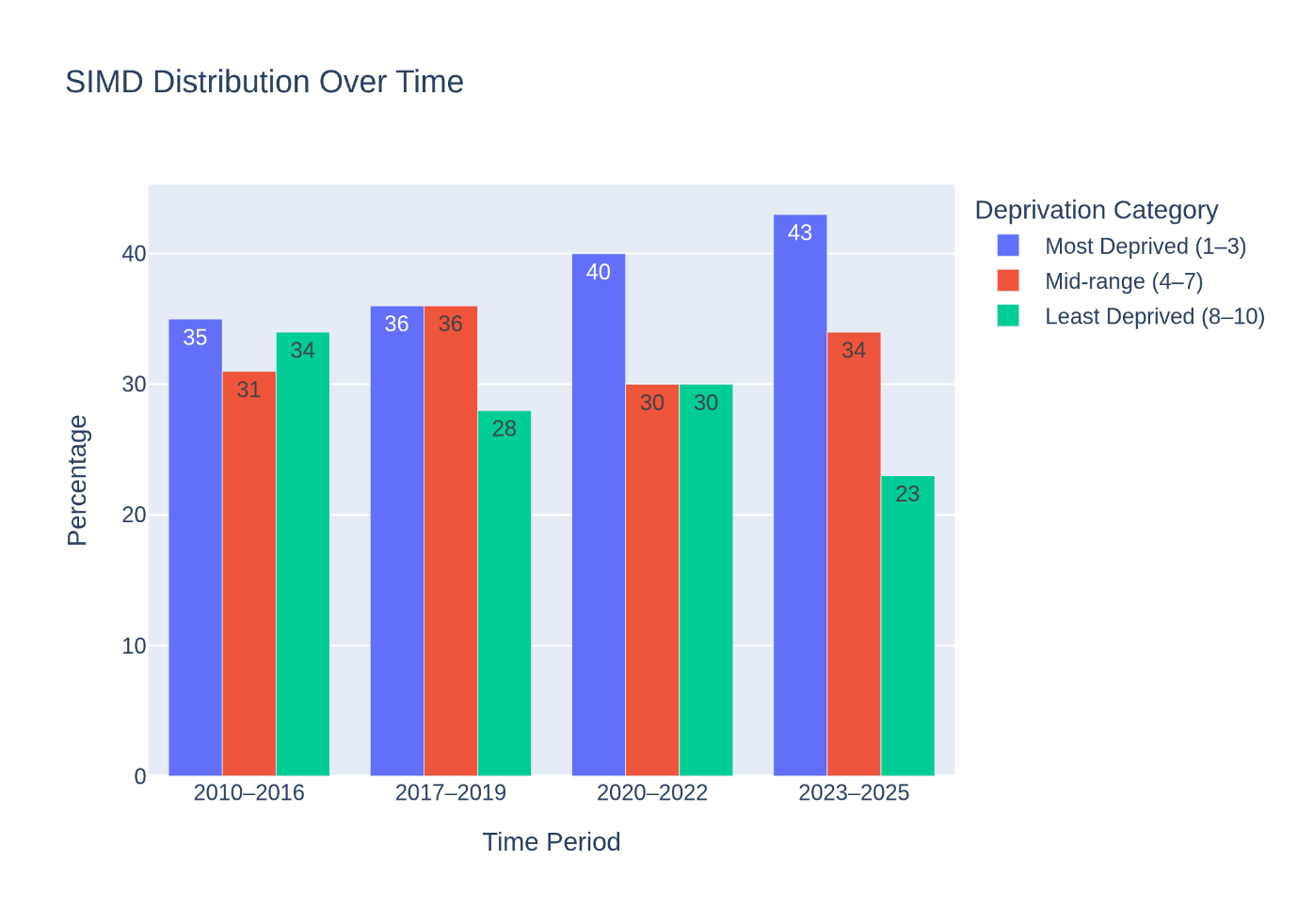


**Supplementary Figure 1:** SIMD distribution


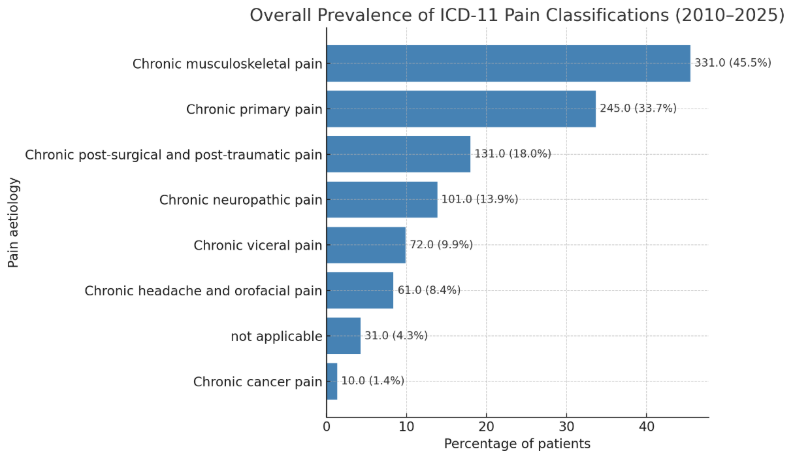


**Supplementary Figure 2:** ICD-11 classification for all patients. Patients with only headache and orofacial pain were seen between 2010-2013, and after that all these patients were diverted to a headache clinic.


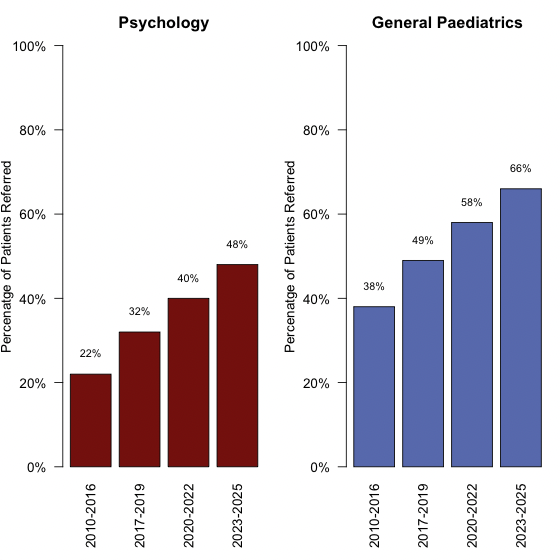


**Supplementary figure 3a:** Significant changes in referrals noted in Primary Chronic Pain Patients


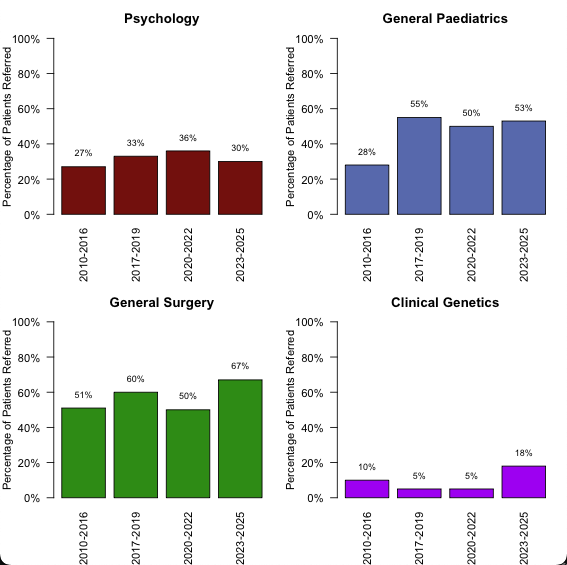


**Supplementary figure 3b:** Significant changes in referrals noted in Secondary Chronic Pain Patients


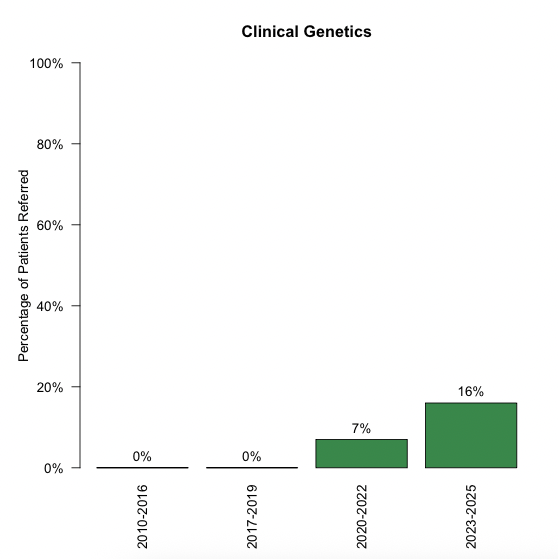


**Supplementary figure 3c:** Significant changes in referrals noted in Mixed Chronic Pain Patients


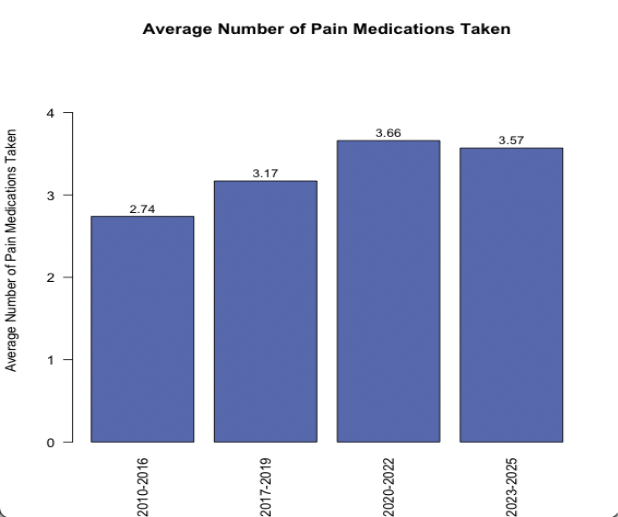


**Supplementary Figure 4a** Mean number of medications taken by paediatric patients between 2010-2025


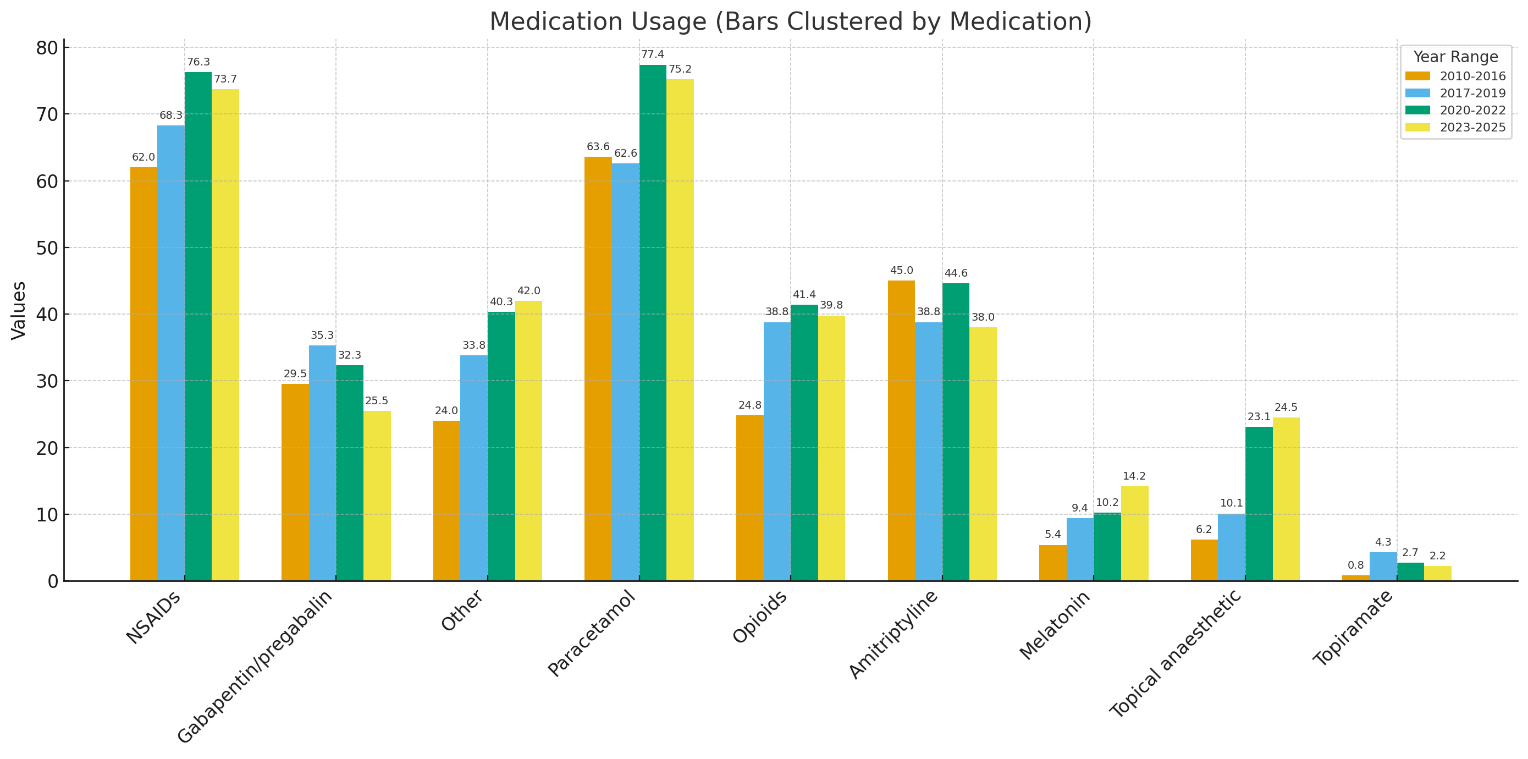


**Supplementary Figure 4b:** The percentage of patients within an epoch taking a certain medication


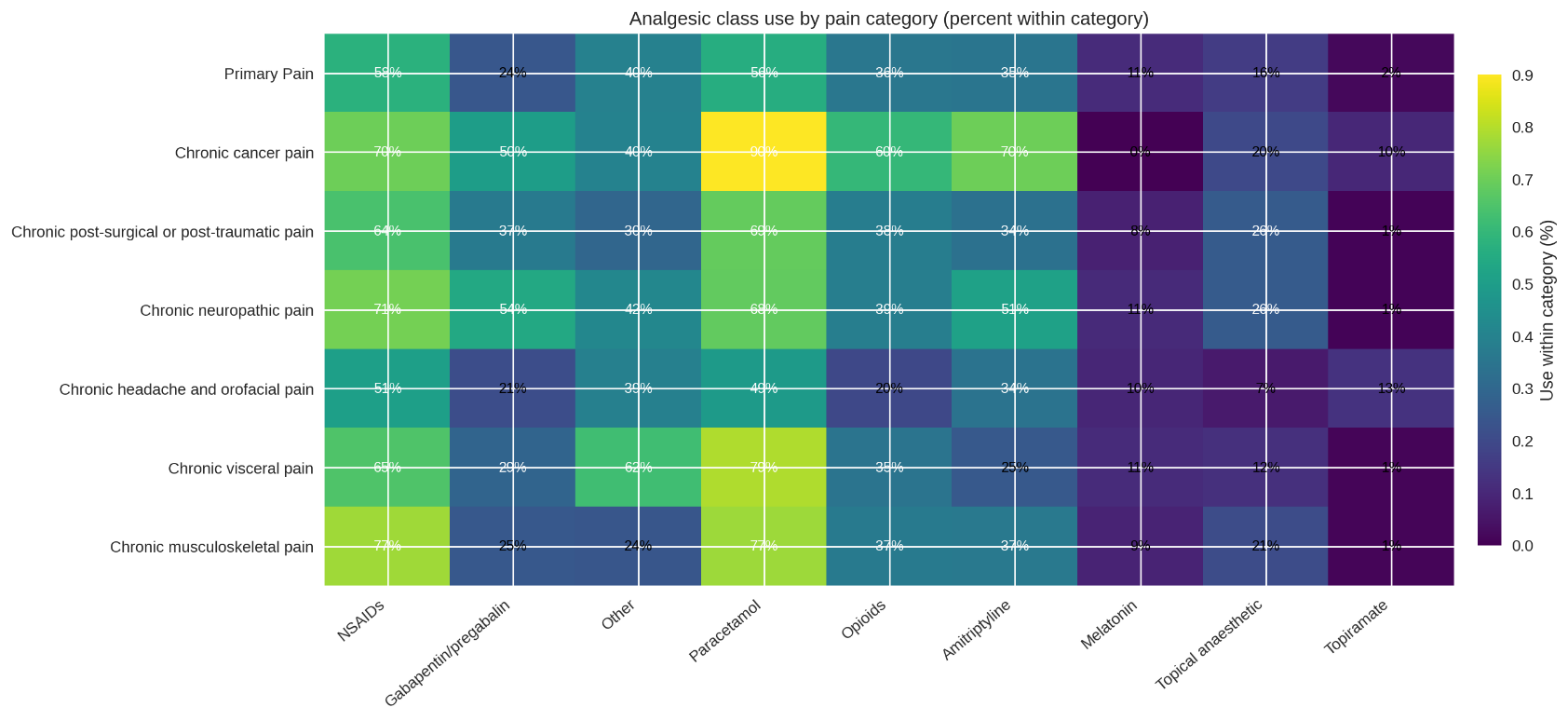


**Supplementary figure 5:** Heatmap of medication usage by pain type


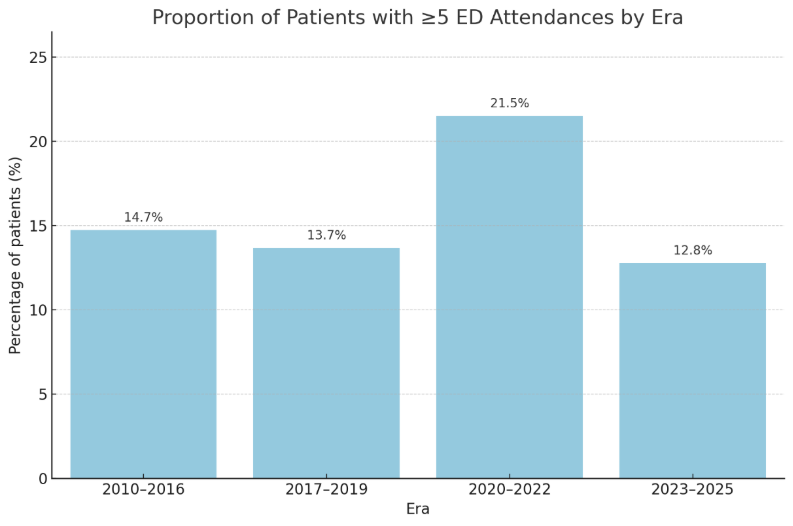


**Supplementary Figure 6**: Percentage of patients with ED attendances greater than or equal to 5


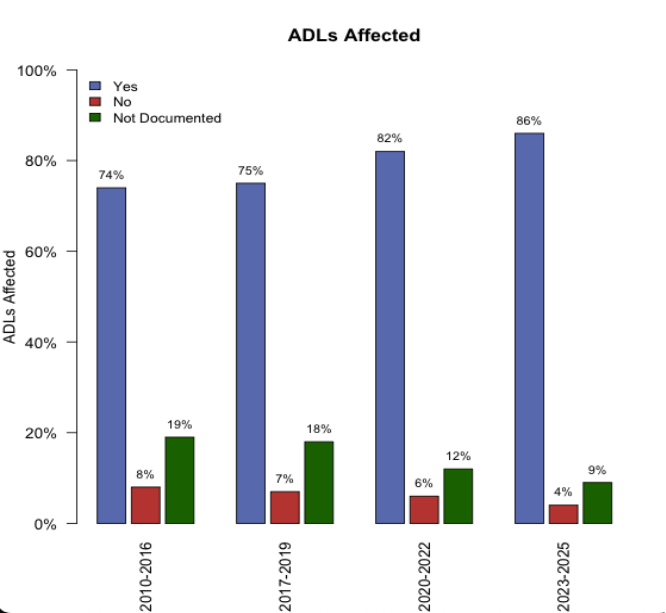


**Supplementary figure 7:** Functional Impact of Pain by Year


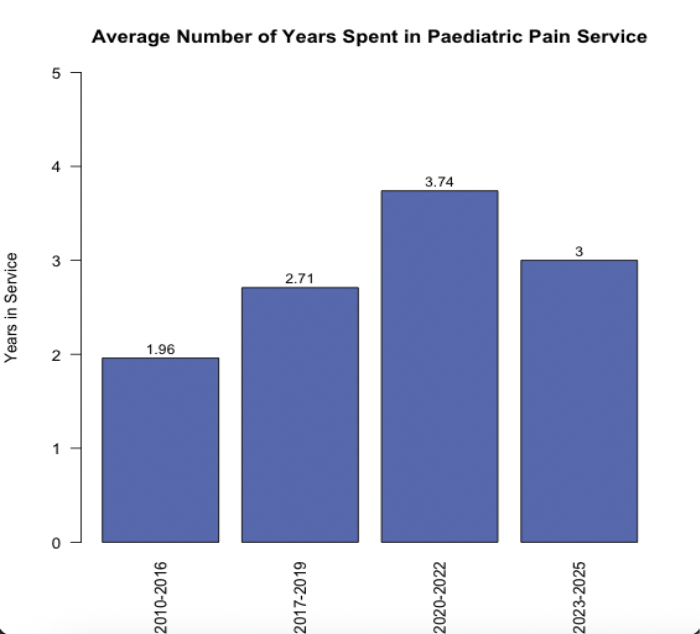


**Supplementary figure 8**: Number of years patients spent with the pain service.

*Supplementary Tables*

| **Reason** | **Number** |
| --- | --- |
| Did not attend | 13 |
| Patient deceased before 1^st^ appointment | 1 |
| No referral and no pain clinic appointment documented | 22 |
| Referral but no appointment documented | 16 |
| Non engagement with service /parent cancelled appointment | 3 |
| Appointment no longer needed (before 1^st^ appointment) | 7 |
| Yet to have 1^st^ appointment - on waiting list | 1 |
| Reviewed in hospital, no further appointments | 1 |
| **Total** | 64 |

**Supplementary Table 1:** Reasons for exclusion

| Pain Type | Total Referrals | GP referrals |
| --- | --- | --- |
| Primary Chronic Pain | Across the periods 2010-2016, 2017-2019, 2020-202, and 2023-2025, patients with a diagnosis of primary chronic pain were referred an average of 7.31 times *(min: 1, max: 30 , SD: 8.09),* 12.32 times *(min: 0, max: 40 , SD: 9.41),* 15.37 times *(min: 1, max: 70 , SD: 12.43),* and 13.72 times *(min: 0, max: 71, SD: 11.20)* respectively. The number of referrals per patient increased by 6.40 between 2010-2016 and 2022-2025 (p: 0.00) | Patients were referred by their general practitioner to a secondary or tertiary service an average of 4.78 times *(min: 0, max: 23 , SD: 5.68),* 6.07 times *(min: 0, max: 32 , SD: 6.66),* 5.32 times *(min: 0, max: 18, SD: 4.71),* and 6.16 times *(min: 0, max: 26, SD: 5.75)* respectively. There was an insignificant increase of 1.38 GP referrals per patient between 2010-2016 and 2022-2025 (p: 0.23). |
| Secondary Chronic Pain | Across the periods 2010-2016, 2017-2019, 2020-202, and 2023-2025, patients with a diagnosis of secondary chronic pain were referred and average of 10.34 times *(min: 0, max: 63 , SD: 9.40),* 15.48 times *(min: 1, max: 124, SD: 17.96),* 13.42 times *(min: 0, max: 67 , SD: 10.77),* and 16.22 times *(min: 1, max: 82, SD: 13.69)* respectively. The number of referrals per patient increased by 5.88 between 2010-2016 and 2022-2025 (p: 0.00) | Patients were referred by their general practitioner to a secondary or tertiary service an average of 4.18 times *(min: 0, max: 26 , SD: 4.89),* 5.55 times *(min: 0, max: 31 , SD: 5.66),* 5.59 times *(min: 0, max: 20, SD: 5.20),* and 5.51 times *(min: 0, max: 55, SD: 8.28)* respectively. There was an insignificant increase of 1.33 GP referrals per patient between 2010-2016 and 2022-2025 (p: 0.15). |
| Mixed Chronic Pain | Across the periods 2010-2016, 2017-2019, 2020-202, and 2023-2025, patients with a diagnosis of mixed chronic pain were referred and average of 11.29 times *(min: 1, max: 28 , SD: 10.26),* 13.20 times *(min: 2, max: 140, SD: 11.84,* 15.52 times *(min: 2, max: 36 , SD: 11.42),* and 12.50 times *(min: 3, max: 51, SD: 12.50)* respectively. There was an insignificant increase of 6.68 referrals per patient between 2010-2016 and 2022-2025 (p: 0.07). | Patients were referred by their general practitioner to a secondary or tertiary service an average of 3.21 times *(min: 0, max: 10 , SD: 3.14),* 4.60 times *(min: 0, max: 20, SD: 5.38),* 6.14 times *(min: 0, max: 22, SD: 6.24),* and 6.31 times *(min: 0, max: 33, SD: 6.51)* respectively. There was an increase of 3.10 GP referrals per patient between 2010-2016 and 2022-2025 (p: 0.04). |

**Supplementary Table 2:** Referral data by pain type

| **Medication** | 2010-2016 (%) | 2017-2019 (%) | 2020-2022 (%) | 2023-2025 (%) | Significance |
| --- | --- | --- | --- | --- | --- |
| NSAIDs | 62 | 68 | 76 | 74 | p = 0.027* |
| Gabapentinoids | 29 | 35 | 32 | 25 | p = 0.180 |
| Paracetamol | 64 | 63 | 77 | 75 | p = 0.002* |
| Opioids | 25 | 39 | 41 | 40 | p = 0.368 |
| Amitriptyline | 45 | 39 | 45 | 38 | p = 0.013* |
| Melatonin | 5 | 9 | 10 | 14 | p = 0.054 |
| Topical Anaesthetic | 6 | 10 | 23 | 24 | p *<0.001** |
| Topiramate | 1 | 4 | 3 | 2 | p = 0.305 |
| Other | 24 | 34 | 40 | 42 | p = 0.003* |

**Supplementary Table 3:** Proportion of patients taking medication for pain. Examples of other medications included: diazepam, lamotrigine, hyoscine butylbromide, baclofen.

| **Social Factor** | 2010-2016 (%) | 2017-2019 (%) | 2020-2022 (%) | 2023-2025 (%) | Significance (Excluding Not Documented) | Significance (Including Not Documented) |
| --- | --- | --- | --- | --- | --- | --- |
| Bedbound | 12 | 16 | 16 | 14 | p=0.000* | p=0.000* |
| Walking Aid | 25 | 24 | 27 | 33 | p=0.000* | p=0.000* |
| ADLs Affected | 74 | 75 | 82 | 86 | p=0.201 | p=0.049* |

**Supplementary Table 4:** Documentation of ADLs affected by Pain

| Patient Name |  | CHI |  | Date |  |
| --- | --- | --- | --- | --- | --- |

HPC-

- Primary diagnosis-
- Duration of pain-
- Pain aetiology-
- Service referred from-

PMH-

- Neurodivergence-
- Mental health/ mood-
- Obesity-
- Hypermobility-
- Sleep

FHx of chronic pain-

DHx-

- Current medications-

- Management tried:

| NSAIDS |  |
| --- | --- |
| Amitriptyline |  |
| Codeine |  |
| Gabapentin/ pregabalin |  |
| Morphine |  |
| Local anaesthesia or nerve blocks |  |
| TENS machine |  |
| Psychological therapy |  |
| Exercise therapy |  |

Social Hx

- Bullying-
- Schooling-
- ACEs-

Plan-

Resources signposted to-

**Supplementary Table 5**: pain clinic pro-forma
